# Re-Evaluation of a Bacterial Antifreeze Protein as an Adhesin with Ice-Binding Activity

**DOI:** 10.1371/journal.pone.0048805

**Published:** 2012-11-07

**Authors:** Shuaiqi Guo, Christopher P. Garnham, John C. Whitney, Laurie A. Graham, Peter L. Davies

**Affiliations:** Protein Function Discovery Group and the Department of Biomedical and Molecular Sciences, Queen's University, Kingston, Ontario, Canada; University of Osnabrueck, Germany

## Abstract

A novel role for antifreeze proteins (AFPs) may reside in an exceptionally large 1.5-MDa adhesin isolated from an Antarctic Gram-negative bacterium, *Marinomonas primoryensis*. *Mp*AFP was purified from bacterial lysates by ice adsorption and gel electrophoresis. We have previously reported that two highly repetitive sequences, region II (RII) and region IV (RIV), divide *Mp*AFP into five distinct regions, all of which require mM Ca^2+^ levels for correct folding. Also, the antifreeze activity is confined to the 322-residue RIV, which forms a Ca^2+^-bound beta-helix containing thirteen Repeats-In-Toxin (RTX)-like repeats. RII accounts for approximately 90% of the mass of *Mp*AFP and is made up of ∼120 tandem 104-residue repeats. Because these repeats are identical in DNA sequence, their number was estimated here by pulsed-field gel electrophoresis. Structural homology analysis by the Protein Homology/analogY Recognition Engine (Phyre2) server indicates that the 104-residue RII repeat adopts an immunoglobulin beta-sandwich fold that is typical of many secreted adhesion proteins. Additional RTX-like repeats in RV may serve as a non-cleavable signal sequence for the type I secretion pathway. Immunodetection shows both repeated regions are uniformly distributed over the cell surface. We suggest that the development of an AFP-like domain within this adhesin attached to the bacterial outer surface serves to transiently bind the host bacteria to ice. This association would keep the bacteria within the upper reaches of the water column where oxygen and nutrients are potentially more abundant. This novel envirotactic role would give AFPs a third function, after freeze avoidance and freeze tolerance: that of transiently binding an organism to ice.

## Introduction

Antifreeze proteins (AFPs) were initially characterized in marine fishes [1], [2] where they protect their hosts from freezing by binding to, and preventing the growth of, seed ice crystals [3]. AFPs lower the freezing temperature of a solution containing ice below the melting point of the ice. This difference between the freezing and melting temperatures is called thermal hysteresis (TH) and is used as a measure of antifreeze activity. AFPs were subsequently found in freeze-tolerant organisms [4], [5] where, rather than preventing freezing, they stop ice crystals in frozen tissues from growing larger through the process of ice recrystallization (IRI) [6].

The bacterium *Marinomonas primoryensis*, isolated from a brackish, ice-covered lake in Antarctica, produces an exceptionally large protein (ca. 1.5 MDa) with Ca^2+^-dependent antifreeze activity [7], [8]. The protein contains two highly repetitive segments, Regions II and IV (RII and RIV), that divide it into five distinct regions (RI–V) [7]. RIV, which contains thirteen 19-aa repeats in tandem, comprising ∼2% of the entire protein, is the only region with antifreeze activity. We recently solved the X-ray crystal structure of *Mp*AFP Region IV to 1.7 Å [9]. This segment of the protein folds as an extended, Ca^2+^-bound right-handed beta-helix whose ice-binding site (IBS) consists of a flat, repetitive array of outward-projecting Thr and Asx residues. The IBS organizes water molecules into a regular ice-like lattice that matches, at a minimum, the primary prism and basal planes of ice. Experimental observation of these ordered surface waters provided strong physical evidence for a mechanism of ice binding that was originally predicted by molecular modeling [10], [11], [12], [13]. This may well be a general mechanism of action for all ice-binding proteins where the IBS orders surface waters into an ice-like “anchored clathrate” pattern that then helps “freeze” the AFPs to the ice surface [9].

Both the TH and IRI activities of AFPs are concentration dependent. Fish typically produce 10–20 mg/ml of AFPs to depress their freezing by the ∼1°C needed to survive in ice-laden seawater [14]. Although fusion proteins can be effective AFPs if the added domain does not occlude the IBS [15], from the perspective of biological efficiency, AFPs are typically small, single domain proteins produced from large multi-gene families. Only in this way can the host produce the millimolar AFP concentrations needed to prevent freezing. It is telling, therefore, that the AFP-active RIV makes up only 2% of the residues in the whole protein. This strongly suggests the primary function of the protein is not that of TH or IRI. To better understand the function of *Mp*AFP, we have examined other regions of the protein.

Here we report the purification and characterization of wild-type *Mp*AFP. Two lambda clones, one from each end of the gene, were sequenced, to reveal both the flanking genes and the domain structure of the AFP. We used Southern blotting of pulsed-field gel separated DNA to ascertain the full extent of RII, and show it makes up more than 90% of the mass of this incredibly large protein. We have derived partial or complete homology models for each of the five regions of *Mp*AFP consistent with its role as an adhesin secreted via its RTX repeats. Using immunofluorescence we have confirmed the location of the protein on the outer surface of the host bacterium, and suggest that mutation and amplification of tandem RTX repeats within Region IV of the adhesin has formed the AFP-like domain that serves to dock the bacterium to ice. This suggests a novel function for an AFP: that of simply binding to ice, rather than preventing its growth or recrystallization.

## Materials and Methods

### MpAFP purification

*M. primoryensis* was cultured and the crude lysate prepared as previously described [8], with the exception that the lysis buffer contained 25 mM Tris-HCl (pH 8.0) and 20 mM CaCl_2_. The soluble portion of this lysate was adjusted to 70% ammonium sulfate then centrifuged at 13,000× g for 30 min at 4°C. The precipitate was resuspended in lysis buffer (50 ml) and dialysed against the same buffer. This material was subjected to ice affinity purification (IAP) [16]. Ice was slowly grown on a cold finger held at −0.5°C for 30 min, then the temperature was gradually decreased to −2.5°C over 48 h until ∼50% of the original volume was frozen. The ice fraction was melted and adjusted to 25 mM Tris-HCl (pH 8.0) and 20 mM CaCl_2_, before being subjected to a second round of IAP, as above. The second ice fraction was then concentrated to 2 ml by dry dialysis in 3,500 molecular weight cut-off dialysis tubing exposed to PEG 8000. This concentrate was then analyzed by standard PAGE under both native and denaturing conditions and the AFP detected using either the cationic carbocyanine dye “Stains-All” (Sigma) or Coomassie blue. Stains-All has been shown to stain Ca^2+^-binding proteins dark blue or purple while staining other proteins red or pink [17].

### Tandem mass spectrometry analysis

Pure *Mp*AFP was resolved by standard SDS-PAGE (10% (w/v) stacking and 4% (w/v) resolving gels) and visualized by Coomassie blue staining. The AFP band was excised and the gel plugs were trypsin digested on a Waters MassPREP station using method 5.7S with reduction (dithiothreitol) and alkylation (iodoacetamide) of any cysteines that might be present. Approximately 200 fmol of tryptically-digested material was fractionated using a Waters CapLC liquid chromatography system with a LC-Packings 75 µm pep-map C18 column. This column was attached directly to the nano Z spray source of a Q-TOF Ultima GLOBAL (Waters Corporation) mass spectrometer and eluted with acetonitrile in 0.1% (v/v) formic acid. An initial TOF-MS survey scan was acquired over the range m/z 400–1600 (from which tryptic fragment masses were determined), and the Q-TOF was programmed to ignore singly charged ions while collecting MS-MS data on up to three co-eluting species. The spectra were smoothed, converted to a list of m/z centroids and submitted to the MASCOT (www.matrixscience.com) and Proteinlynx Global Server 2.0 (www.waters.com) search engines for database comparison. Those peaks that were not identified as trypsin or keratin were manually sequenced.

### Genomic DNA extraction

The CTAB method of chromosomal DNA extraction [18] was used on a 50-ml culture of *Marinomonas primoryensis* grown for 5 days at 4°C in 50% (w/v) SWB (19 g/l sea salt (Sigma); 1 g/l Tryptone; 1 g/l yeast extract) as above. This DNA was used in subsequent PCR reactions and in the construction of a genomic library.

### Amplification of a fragment of MpAFP sequence

Two fully-degenerate primers were designed based on amino acid sequences determined above. The sense primer 5′-GAYGCNACNTTYGARGCNGCNAA-3′ corresponds to DATFEAAN. The antisense primer 5′-TCRTCRTTNCCNGTNCCNGCRTC-3′ corresponds to DAGTGNDE. PCR conditions using 3 µM of each primer were as follows: 30 cycles of 95°C for 30 s, 50°C for 1 min and 72°C for 90 sec with a final extension at 72°C for 8 min. The resulting product was purified by gel extraction (Qiagen gel extraction kit), cloned using the TOPO TA kit (Invitrogen), and sequenced at the Cortec DNA Service Laboratories, Kingston, Ontario. Additional sequence was obtained by inverse PCR but ultimately, a more complete sequence was obtained as described below.

### Genomic Lambda library construction and analysis

A genomic Lambda Dash II library was constructed from *M. primoryensis* DNA partially digested with *Sau3A*I (BioS&T, Montréal, Canada). It afforded ∼16-fold coverage of the genome as it had a titre of 4×10^6^ pfu/ml with an average insert size of 20 kb. A PCR product, corresponding to bases 3634–4053 of GenBank Accession ABL74377, was labeled with [α^32^P]-dCTP using a random priming DNA labeling system (Invitrogen, Carlsbad, California) and was used to screen the library by standard methodologies [19]. Phage DNA was isolated by the CsCl gradient technique [18]. DNA insert size was determined by digestion with *Sal*I. After this clone was sequenced (below) it was necessary to isolate a second clone to obtain the 5′ end of the *Mp*AFP gene. A PCR product corresponding to two repeats from the highly-repetitive section RII found at the end of the first clone was amplified (bases 1946–2569 of Genbank Accession ABL74378. A clone which hybridized to this probe, but not to the probe used earlier, was isolated.

### Sequencing of lambda clones

Lambda phage DNA was mechanically sheared and shotgun cloned into pUC19 vector (Genome Québec, McGill University, Montréal, Canada). A total of 288 randomly selected clones were initially sequenced using the M13 forward primer. Gaps in the sequence were closed by sequencing relevant clones with either the M13 reverse primer or by sequence walking. In total, 390 sequence reads were performed to ensure double coverage of all regions except the highly repetitive region.

### Pulsed-field gel electrophoresis

The CHEF Bacterial Genomic DNA Plug Kit (catalog 170–3592) (Bio-Rad laboratories, Hercules, California) was used to prepare agarose plugs containing *M. primoryensis* for in-gel restriction endonuclease digestion. The kit was used according to manufacturer's instructions except that the cells were resuspended in a higher salt buffer (10 mM Tris-HCl (pH 7.2), 330 mM NaCl, and 150 mM EDTA (pH 8.0)) prior to agarose addition. Digests were also performed according to kit instructions using the restriction enzymes *Pst*I, *Ase*I, *Mse*I, *Alu*I and *Msp*I (New England Biolabs). After washing the plugs in gel buffer (0.5X TBE), they were embedded in a 20-cm-long 1% (w/v) agarose gel. The gel was run in a CHEF-DR® II Pulsed Field Electrophoresis System (Bio-Rad laboratories, Hercules, California) at 120 V for 22 h with a linearly ramped 50–90 s switch time during the length of the run. The temperature was maintained at ca. 4°C. The gel was stained with a 1 µg/ml solution of ethidium bromide to allow visualization of DNA samples and standards.

### Southern blotting

The PFGE gel from above was blotted onto a Zeta-Probe® membrane (Bio-Rad laboratories, Hercules, California) by the alkaline capillary method [18] and probed with the repeat-containing fragment as above. After washing to remove excess probe, Kodak BioMax XAR film was exposed to the membrane for 16 h.

### Homology modeling of MpAFP domains

Sections of the *Mp*AFP sequence were submitted to the Phyre2 server [20]. Phyre2 uses the hidden Markov method to generate alignments of a submitted protein sequence against proteins with published structures [21]. The resulting alignments are then used to produce homology-based models of the query sequence to predict its three-dimensional structure. In addition, Phyre2 uses an *ab intio* folding simulation called Poing to model regions of a query with no detectable similarities to known structures [22]. Poing combines multiple templates of known structures to produce the final model of the query sequence. The model is judged to be accurate when over 90% of the submitted residues are modeled at greater than 90% confidence [20].

### Production of polyclonal antibodies to MpAFP RII and RIV

Two recombinant proteins, corresponding to RII (beginning at residues TTGS and ending at GNTVD) and RIV (beginning at residues NVSQ and ending at MVTV) from *Mp*AFP (Genbank ABL74378.1) were produced in *E. coli* with N-terminal His_6_-tags. Once the His-tags were removed via thrombin cleavage, aliquots (750 µg) were emulsified using TiterMax® (Cedarlane, Burlington, Canada) and used as separate antigens for the production of polyclonal antibodies. Single doses were injected into rabbits, and sera were collected approximately 6 weeks later.

### Immunodetection and fluorescence microscopy imaging of MpAFP

An aliquot (0.5 mL) of an *M. primoryensis* culture in its stationary growth phase (OD_600_ = 1.3) was centrifuged at 2,000× g for 10 min. The cell pellet was resuspended in 1 ml of 0.85% (w/v) NaCl and an aliquot (10 µl) was pipetted onto a round coverslip. The cells were air dried for 30 min then fixed in 1% (v/v) paraformaldehyde for 20 min. After three 10-min washes in 0.85% (w/v) NaCl, the coverslips were incubated with a 1∶200 dilution of anti-sera against either *Mp*AFP_RII or RIV in the same solution at room temperature for 1 h. After three washes as above, the coverslips were incubated in the dark with a 1∶200 dilution of goat anti-rabbit Alexa Fluor 350 secondary antibodies (Invitrogen) for 1 h at room temperature. To test the specificity of the secondary antibody, a control experiment was also carried out in which the fixed cells were incubated with fluorescent secondary antibodies in absence of the primary antibodies. After three more washes, coverslips were incubated in the dark with 0.05 mM SYTO 9 (Invitrogen) for 30 min to stain DNA. After three final washes, mounting medium (DAKO) was used to fix the sample onto a slide. The medium was allowed to solidify in the dark overnight. Images were obtained using a Quorum Wave FX Spinning Disc confocal fluorescent microscope system equipped with a Hamamatsu Orca camera. The images were edited using Metamorph software. A control experiment with *E. coli* was also conducted. The cells were grown overnight at 37°C (OD_600_ = 1.4) in LB broth Miller (EMD) and the procedures were repeated as above.

## Results

### MpAFP is an exceptionally large protein

**Figure 1:**
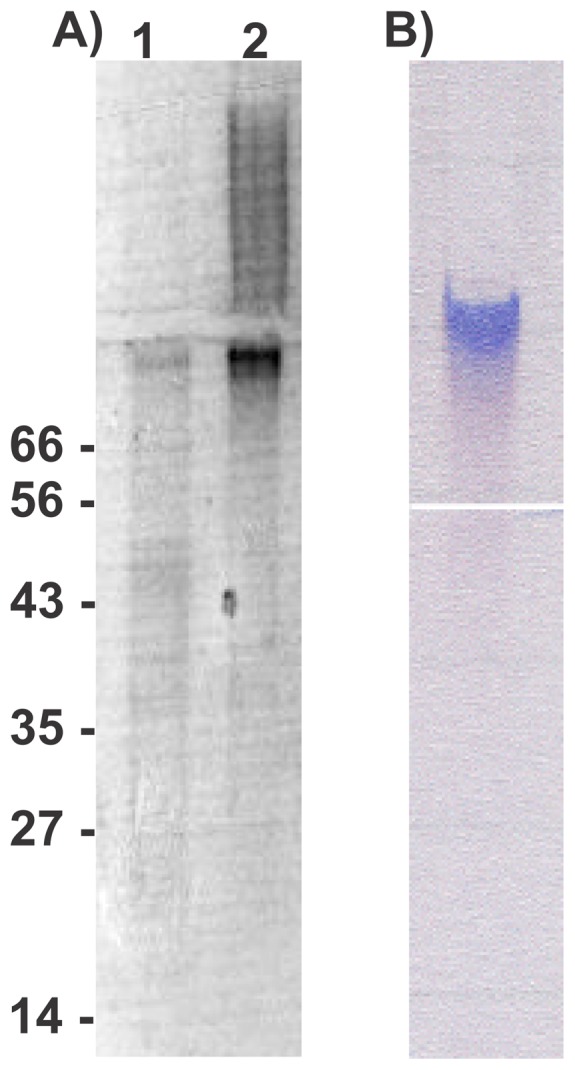
Polyacrylamide gel electrophoresis of *Mp*AFP-enriched fractions. (A) The ice-bound fractions from one (lane 1) and two (lane 2) cycles of ice affinity purification were subjected to SDS-PAGE and stained with Coomassie blue. *Mp*AFP barely entered the resolving section of the gel. The locations of molecular mass markers (kDa) are indicated on the left. A right-pointing arrow indicates the junction between the stacking and resolving. (B) The ice-bound fraction from the second cycle of ice affinity purification was electrophoresed on a non-denaturing gel and stained with Stains-all. *Mp*AFP was still present within the stacking section of the gel when the bromophenol blue marker had reached the bottom of the separating gel. A left-pointing arrow indicates the junction between the stacking and resolving gels.

Ion-exchange and gel-permeation chromatographies were ineffective at purifying *Mp*AFP from crude bacterial lysate. A series of peaks showing only low antifreeze activity eluted from DEAE-cellulose at >0.5 M NaCl and these were contaminated with nucleic acids (not shown). Active material failed to elute during size-exclusion chromatography, despite the use of three different column matrices (not shown). In place of column chromatography, ammonium sulfate precipitation followed by two cycles of ice-affinity purification (IAP) were used to purify *Mp*AFP. In the latter technique, only the proteins that bind to ice are readily incorporated into a slowly grown ice mass. When the ice-bound material was analyzed by SDS-PAGE, there was a marked enrichment of a very high molecular weight band after the first round of IAP, which became the major protein present after the second cycle (Fig. 1A). This protein barely entered the 10% (w/v) resolving gel. When electrophoresed on a non-denaturing polyacrylamide gel (Fig. 1B), the protein remained within the 4% (w/v) stacking gel and stained intensely with Stains-All, a dye that is particularly effective at visualizing Ca^2+^-binding proteins. The equivalent region from an unstained native gel was mashed in buffer and showed thermal hysteresis activity of ∼0.4°C.

### MpAFP is a bacterial Repeat-in-ToXin (RTX) protein

Following digestion of the gel-purified *Mp*AFP with trypsin, a peptide fingerprint was generated and a number of peptides were subjected to LC-MS/MS spectrometry (Table S1). A representative example of the tandem mass spectrometric sequencing is presented for the 1408.6-Da peptide IDAGTGNDEIYIK (Fig. S1). The complete y-ion series was evident, along with some y^o^ (-H_2_O), a- and b-series ions. BLAST searches indicated that many of the sequenced peptides showed similarity to proteins from the RTX family of virulence factors, including one that was 69% identical to a sequence from *Saccharophagus degradans* 2–40 (GI:89950541). Since the peptide above, as well as the peptide EADATFEAANISYGR (Table S1), mapped 418 residues apart on the *S. degradans* RTX protein, they were used to design degenerate primers from which a ∼1-kb segment of the *Mp*AFP gene was amplified.

### MpAFP is a multi-domain protein with five distinct regions

**Figure 2:**
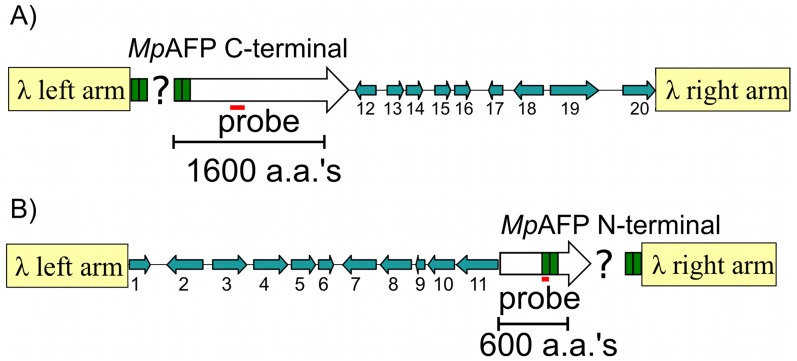
Lambda library clones encoding *Mp*AFP. (A) Schematic layout of the lambda clone containing DNA coding for the C-terminal portion of *Mp*AFP and nine downstream genes, 12–20. The flanking genes (blue arrows) are identified by their numbers in Table S2. The arrows point in the direction of transcription. The left and right arms of lambda phage (not to scale) are indicted by the tan-colored rectangles. Green boxes represent the tandem, identical 312-bp repeats of *Mp*AFP. The question mark represents the unknown number of these repeats present within the clone. The location of the initial hybridization probe is indicated by the horizontal red line. The scale is indicated by the bracket representing DNA coding for 1600 amino acids (aa's). (B) Schematic layout of a second lambda clone that contains DNA coding for the N-terminal portion of *Mp*AFP and eleven upstream genes (1–11). Symbols are as described in (A), with the bracket representing DNA coding for 600 amino acids and the red bar indicates the location of the second hybridization probe.

**Figure 3:**
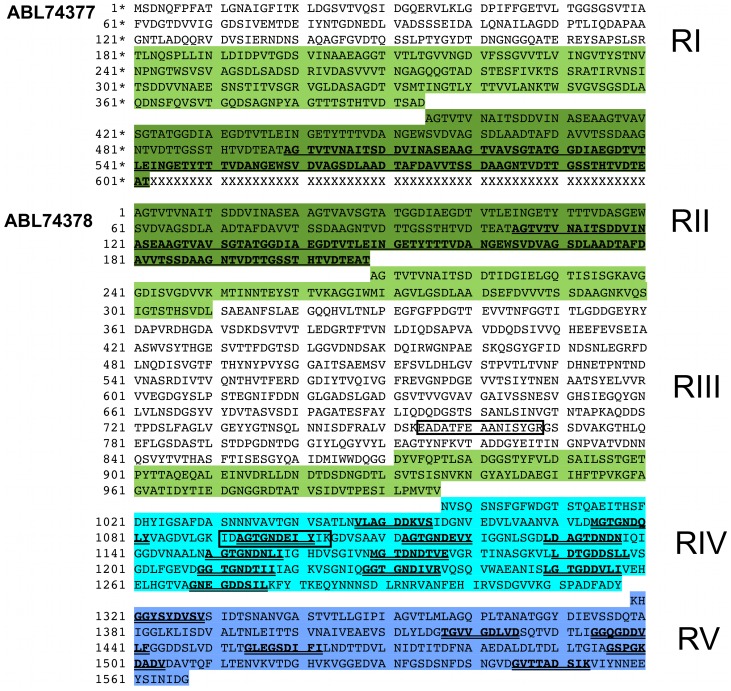
Amino acid sequence of *Mp*AFP. The complete amino acid sequence of *Mp*AFP is divided into five distinct regions (RI–RV). Because it was not possible to sequence through the DNA encoding the highly repetitive RII (∼120 identical 104-aa repeats), the protein was deposited in NCBI with two accession codes: ABL74377 and ABL74378. The two segments of *Mp*AFP are separated by a line of “Xs”. The first segment of *Mp*AFP contains RI and two 104-aa repeats of RII. The residues are identified with asterisks (1*–602*). The second segment begins with two 104-aa repeats of RII and continues through RIII, RIV and RV, with the residues identified by regular numbers (1–1567). The color scheme for the highlighted residues corresponds to that of Fig. 6. The second 104-aa repeat of RII is indicated in bold underlined letters in both segments of *Mp*AFP. The nine-residue RTX-like repeats in RIV and RV are represented by bold double-underlined letters. The boxed sequences EADATFEAANISVGR and IDAGTGNDEIYIK are the two sequenced tryptic peptides identified by tandem MS/MS (Table S1) that were used to design degenerate PCR primers that amplified the first nucleic acid probe used for the isolation of the *Mp*AFP gene.

The first lambda Dash II clone was isolated by screening the *M. primoryensis* genomic DNA library with a probe corresponding to a C-terminal portion of the gene (Fig. 2A). The ∼21 kb insert in the phage encoded the C-terminal end of *Mp*AFP and extended for over 10.5 kb into 3′-flanking DNA. *Mp*AFP coding region occupied the other 10.5 kb, but ∼8.5 kb at the 5′ end consisted entirely of a series of identical, tandem 312-bp repeats. To locate the 5′-end of the gene, a phage clone was selected that hybridized to the 312-bp repeat but not to the C-terminal probe. This second clone contained the 5′-end of the gene as well as over 12.5 kb of 5′-flanking DNA for a total insert size of ∼18 kb (Fig. 2B). Like the previous clone, it contained an undetermined number of the 312-bp repeats, but here they spanned ∼4.5 kb at the 3′ end of the insert. A large number of shotgun clones (45 in total) contained these repeats, but as the DNA sequences of all repeats were 100% identical (except the final one which has a single base difference), it was not possible to assemble them into a contiguous sequence. Nevertheless, the two genomic lambda clones revealed the entire sequence of *Mp*AFP, with an undetermined number of 104-aa repeats (Fig. 3), as well as a total of twenty flanking genes (11 upstream and 9 downstream (Table S2)).

*Mp*AFP can be divided into five distinct Regions (I–V) two of which (II and IV) are highly repetitive. Region II contains the 104-aa repeats mentioned above. Region IV is 322 amino acids (aa) long and contains thirteen tandem copies of a low to moderately conserved 19–21 residue repeat with the consensus sequence xGTGNDxuxuGGxuxGxux (where x can be any amino acid and u represents a large hydrophobic residue). We have determined that this region of RTX repeats folds as a Ca^2+^-bound beta-solenoid and behaves like a hyperactive AFP [9]. The remainder of the protein is non-repetitive and consists of the Regions I (394 aa), III (788 aa), and V (249 aa). The two genes that immediately flank the *Mp*AFP gene are a putative sulfate permease (366 bp upstream) and peptide methionine sulfoxide reductase (100 bp downstream). *Mp*AFP is not part of an operon as both of these genes are in an inverted orientation with respect to *Mp*AFP. The promoter for the *Mp*AFP gene contains a well-defined -35 (TTGATT) and -10 (TAATTA) sequence upstream of the putative transcription start site, as well as a putative AGGAGA ribosome binding site 6 bp upstream of the ATG start codon.

### MpAFP contains ca. 120 copies of the 104-aa repeat

**Figure 4:**
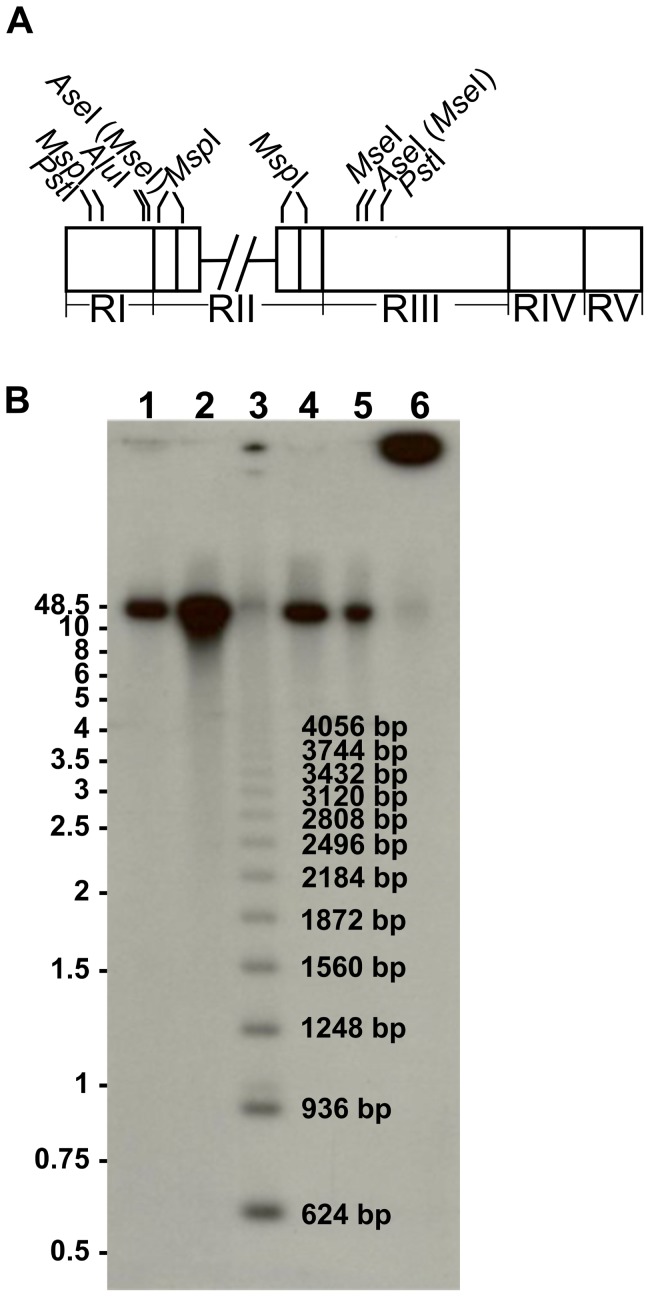
Estimation of 312-bp repeat copy number by Southern blotting. (A) Schematic diagram of the *Mp*AFP coding region illustrating the relative positions of restriction enzyme cut sites. Cross-hatched lines indicate the break between the two segments of *Mp*AFP coding region described in Fig. 3. Four restriction enzymes, including *Mse*I, *Ase*I, *Pst*I and *Alu*I cut only outside the 312-bp repeats. Since the cut site of *Mse*I (T/TAA) is present within that of *Ase*I (AT/TAAT), *Mse*I is indicated in parentheses beside *Ase*I. *Msp*I is the only restriction enzyme in this set that cuts within the 312-bp repeats in RII, and it also cuts once in RI. (B) Southern blot of the digests using a 2×312-bp repeat from RII as the probe. Undigested DNA (lane 6) remained near the well, whereas the four restriction enzymes *Alu*I, *Pst*I, *Ase*I and *Mse*I (lane 1, 2, 4 and 5) that cut outside the repeats produced a fragment of approximately 37,500 bp in length. The *Msp*I (lane 3) partial digest produced a ladder of bands at 312-bp intervals and those containing between 2 (624 bp) and 13 (4056 bp) repeats are marked on this blot. DNA length markers (kb) are indicated on the left of the blot.

To determine the number of 312-bp (104-aa) repeats present within the *Mp*AFP gene, and therefore the total size of *Mp*AFP, in-gel restriction endonuclease digestion of *in situ* lysed *M. primoryensis* bacteria was followed by pulsed-field gel electrophoresis (Fig. 4A, B). Four different enzymes that cut just outside of the repeats but not within it were selected, along with *Msp*I, which cuts once within each repeat (Fig. 4A). Southern blotting using the repeat as a probe showed that undigested DNA remained near the well (Fig. 4B, lane 6) whereas the four restriction enzymes that cut outside the repeats produced a fragment ca. 37,500 bp in length (lanes 1, 2, 4, and 5), equivalent to ∼120 copies of this 312 bp repeat. The *Msp*I partial digest produced a ladder of bands at 312 bp intervals and those containing between 2 and 13 repeats are clearly visible on the blot. This result and the analysis of the lambda clones show that RII contains ∼120 copies of identical 312 bp repeats in tandem. This makes *Mp*AFP a massive protein (ca. 1.5 MDa), with RII accounting for roughly 90% of its size (∼12,480 aa) compared to a total of 1433 aa for the other four regions combined.

### Bioinformatics analysis of MpAFP reveals homologous proteins with similar domain architectures

BLASTp searches performed using *Mp*AFP identified matches to many outer membrane adhesion proteins in Gram-negative bacteria (see Text S1 for details). Moreover, in conserved domain analyses, RII was identified as a poorly characterized repeat found in bacteria (expect value ∼10^−25^, pfam13753), which is similar to the bacterial Immunoglobulin (Ig)-like fold found in a variety of bacterial surface proteins (expect value ∼10^−3^, Pfam PF13754). The second match was between RV and Pfam PF08548 (expect value ∼10^−3^), a serralysin C-terminal domain thought to be important for secretion through the bacterial cell wall via the type I secretion pathway (TISS) [23].

### The homology model of a single 104-aa repeat of MpAFP folds as an immunoglobulin-like beta-sandwich, confirming its identification as an adhesion protein

Structures of proteins with high sequence similarity to *Mp*AFP have not been described, but the conserved domain search suggested that the Protein Homology/analogY Recognition Engine (Phyre 2) might be able to generate homology models. A single 104-aa repeat segment of *Mp*AFP_RII was submitted to the Phyre2 server. It was modeled by the suite of programs as an S-type immunoglobulin (Ig)-like beta-sandwich (Table 1) with seven alphabetically listed beta-strands arranged in a Greek-key topology [24]. The N-terminal A and B strands hydrogen bond with strand E to form sheet I that packs against sheet II consisting of the other four strands (G, F, C and D) (Fig. 6). Although the six template structures for the final model show only ca. 20% sequence identity to the 104-aa RII, 92% of the protein's residues were modeled at greater than 90% confidence.

**Figure 5:**
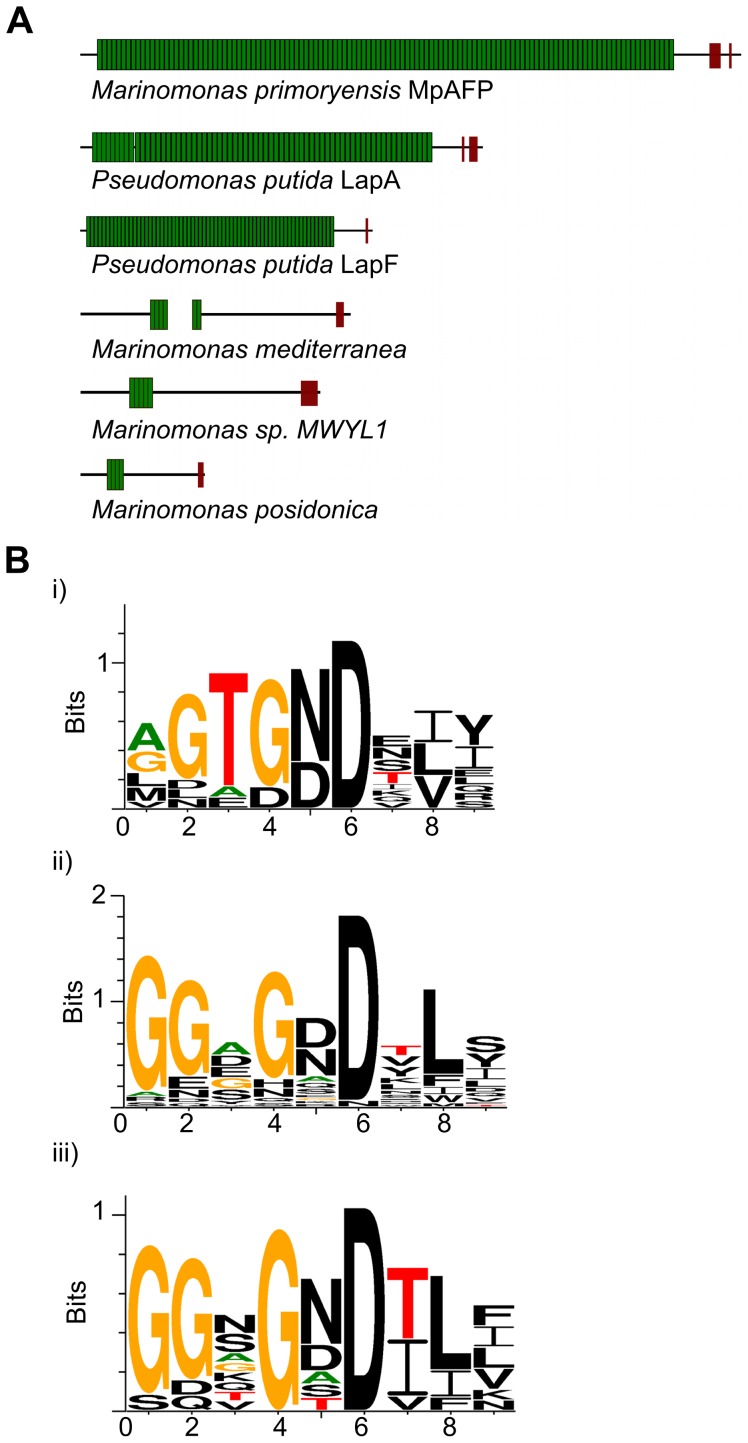
Schematic representations of *Mp*AFP and other large adhesion proteins. (A) Proteins are drawn to scale. Each Ig-like unit of the large repetitive region is shown as a green rectangle. The RTX-repeats in the C-terminal region are illustrated as brown blocks. The schematic diagrams are based on the following sequences from NCBI: *Pseudomonas putida* KT2440 LapA (NP_742337) and LapF (NP_742967), *M. sp. MWYL1* hemolysin-type calcium-binding protein (YP_001340713), *M. posidonica* outer membrane adhesin-like protein (YP_004481992) and the *M. mediterranea* homologues (YP_004313542+YP_004313541) that are separated by one nucleotide preceding an irregular GTG start codon. (B) Weblogo representations of 9-aa RTX repeats. Consensus plots are shown for: i) 12 RTX repeats in *Mp*AFP_RIV; ii) 29 RTX repeats from homologs of *Mp*AFP from three other *Marinomonas* species shown in Fig. 5A; iii) 10 RTX repeats in adhesion proteins LapA and LapF. As shown in the weblogo plots, the RTX repeats from the three homologs of *Mp*AFP and the LAPs follow the consensus of the conventional nine-residue RTX repeats of GGxGxDxUx (Fig. 5Bii and 5Biii). The RTX-like repeats in *Mp*AFP_RIV deviate from the conventional RTX repeats by introducing conserved ice-binding residues at positions 3 and 5 (Thr and Asx, Fig. 5Bi). Residues are colored black except for Gly (orange), Thr (red), and Ala (green).

**Figure 6:**
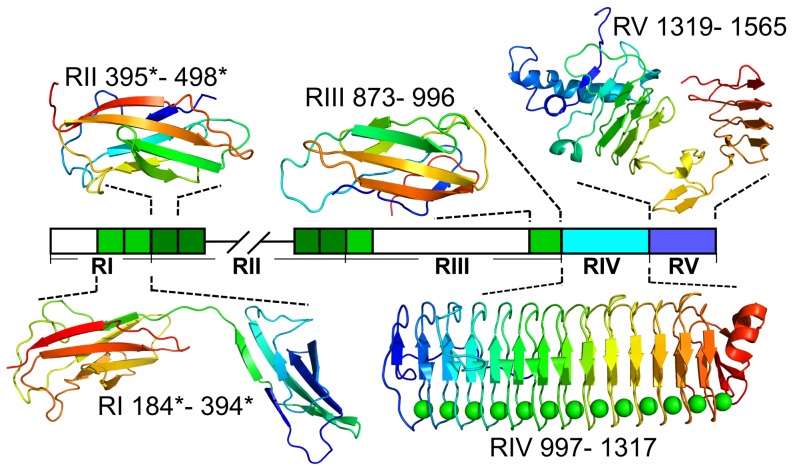
Homology models produced for domains of *Mp*AFP using Phyre2. The linear map of the regions of *Mp*AFP is colored as in Fig. 3. Hatched lines indicate the break between the two contiguously sequenced segments of *Mp*AFP. Uncolored regions could only be modeled *ab initio* by the Phyre2 server and did not produce reliable results. Colored regions were modeled at greater than 90% confidence and their structures in ribbon representation are shown above and below the map of *Mp*AFP. The X-ray crystal structure of RIV is shown [9]. Residues for each model/structure are numbered according to the sequence in Fig. 3. The protein folds are shown in the colors of the rainbow from the N terminus (blue) to the C terminus (red).

**Table 1:** Statistics of homology modeling studies on *Mp*AFP domains.

Regions	Residues modelled at 90% confidence (%)	Templates	Sequence identity to the templates (%)	Organisms
RI (394 aa)	36	Fibronectin (III)-like	15	*Clostridium thermocellum*
				
RII (104 aa)	92			
		Fibronectin (III)-like	20	*Clostridium thermocellum*
		Fibronectin (III)-like	16	*Clostridium thermocellum*
		Fibronectin (III)-like	13	*Clostridium thermocellum*
				
RIII (788 aa)	16			
		Cadherin-1	9	Mouse
		Cadherin-2	15	Mouse
		Ep-cadherin	12	Mouse
				
RV (249 aa)	98			
		I.3 lipase	12	*Pseudomonas sp. Mis38*
		I.3 lipase	13	*Serratia2 marcescens*

The template structures used to model this domain consist of the two divergent repeats from the fibronectin (III)-like module from *Clostridium thermocellum* (PDB accession 3PE9 and 3PDG) and a sulfite oxidase from chicken (PDB: 2A9D). In addition, several other structures also demonstrated high quality structural matches (confidence greater than 80%) to the single repeat of 104-aa RII, including regions of a collagen adhesin from *Staphylococcus aureus* [25]. All of these matches were to regions of these proteins that adopt the Ig-like beta-sandwich fold. Identical results were obtained using the I-TASSER server in place of Phyre2.

### The Ig-like fold also extends into RI and RIII

The repeat motif of RII is also found in the adjoining portion of RI and RIII. However, these three copies (two in RI and one at the C terminus of RII, shown as light green boxes in Fig. 6) are not as well conserved, with only 41–63% identity to the RII repeat (Fig. S2). Not surprisingly, they were also modeled as Ig-like beta-sandwich structures based on fibronectin-like modules from *Clostridium thermocellum* (3PE9 and 3PDG), which was also one of the templates used to model RII. The remainder of RIII shows no sequence similarity to RII and no evidence of any sequence repeats, yet the C-terminal 124-aa portion of RIII was also modeled, at greater than 90% confidence, as an Ig-like beta-sandwich. The templates for the final model were portions of mouse cadherin (E-cadherin ectodomain (PDB: 3Q2V) and N-cadherin ectodomain (PDB: 3Q2W)) which are Ca^2+^-binding transmembrane proteins involved in cell-cell adhesion [26]. All of the additional templates used to model this region adopt an Ig-like beta-sandwich fold and included fibronectin (III)-like modules (eg. PDB: 3PDD), carbohydrate-binding modules (eg. PDB: 2C26) and collagen-binding modules of collagenase (eg. PDB: 3JQU). All of these template proteins are involved in adhesion. The N-terminal 183 aa of RI and the central 587 aa of RIII did not generate models.

### The fold of RV is predicted to include a Ca^2+^-dependent beta-roll

Phyre2 predicted that the 249-aa *Mp*AFP_RV adopts a Ca^2+^-bound beta-roll-containing structure in which 98% of the residues were modeled at over 90% confidence, despite only 13% sequence identity. The final model was constructed using extracellular lipases from *Pseudomonas sp. mis38* (PDB: 2ZJ6) and *Serratia marcescens* (PDB: 2QUB). Both of the lipases belong to the I.3 family and consist of an N-terminal catalytic domain that is rich in alpha-helices and a C-terminal beta-roll containing RTX repeats with Ca^2+^- ions coordinated in the turns. *Mp*AFP_RV was modeled based on the C-terminal domain.

Several other modules also demonstrate excellent structural alignment (>95% confidence) to *Mp*AFP_RV. These include the C-terminal domain of *Serralysin*-like metalloproteases (PDB: 1G9K and 1K7G), a region from the secreted protease C (PDB: 1K7Q) and even the crystal structure of *Mp*AFP_RIV. Again, these are all RTX-like, Ca^2+^-dependent beta-rolls, although models produced by Phyre2 do not include metal ions.

### MpAFP is localized to the cell surface of *M. primoryensis*

Evidence that *Mp*AFP is localized to the exterior of the cell surface includes: 1) the presence of non-cleavable secretion signals near the C terminus (RTX repeats) indicates that *Mp*AFP is secreted via TISS. This secretion system will allow *Mp*AFP to be transported directly through the bacterial membranes without forming periplasmic intermediates. 2) No antifreeze activity is released into the cell culture medium of *M. primoryensis* [8]. This suggests that although *Mp*AFP is secreted via TISS, it remains bound to the cells. 3) Bioinformatics analyses outlined above show that *Mp*AFP has the hallmarks of an outer membrane adhesion protein. 4) Circular dichroism analyses demonstrate that region IV of *Mp*AFP will only take on its beta-rich structure in the presence of the millimolar Ca^2+^ levels found in the bacterium's natural environment [8]. The other regions of *Mp*AFP also show dependence on millimolar Ca^2+^ for folding, and are predominantly random coil in the presence of excess EDTA (data not shown). The low cytosolic Ca^2+^ concentration (high nanomolar range) is insufficient to fold *Mp*AFP, thus eliminating the possibility of it being functional inside the cell.

**Figure 7:**
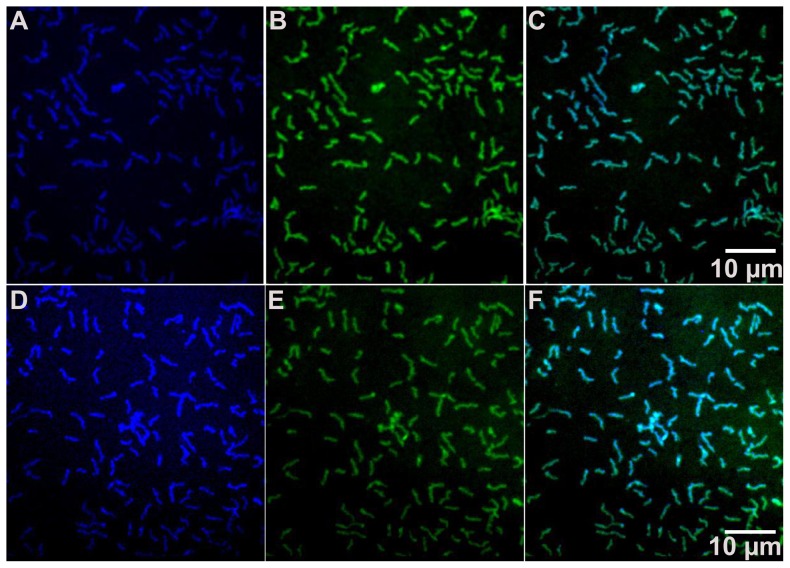
Localization of *Mp*AFP by immunofluorescence. Immobilized *M. primoryensis* cells were fixed in 1% paraformaldehyde before being incubated with anti-RII (A, B and C) or anti-RIV (D, E and F) polyclonal antibodies, followed by Alexa Fluor 350 - conjugated goat anti-rabbit secondary antibody (blue in A and D) and SYTO 9 (green in B and E). Images (C) and (F) are composite images of (A) and (B), and (D) and (E), respectively. A 10-micron scale marker is shown by the white horizontal line in panels C and F.

Here we have also used immunodetection to confirm the localization of *Mp*AFP on the cell surface. We used antisera specific for RII or RIV, followed by a secondary antibody conjugated to a blue fluorophore, to screen for the presence of *Mp*AFP on intact whole cells affixed to coverslips. The cells were counterstained with the cell-permeable nucleic acid dye SYTO 9 to differentiate the bacteria from debris. The green fluorescence of the nucleic acid stain clearly showed the bacteria as rod-shaped cells 2–3 µm in length (Fig. 7B and 7E). Blue fluorescence, indicating the presence of *Mp*AFP, was co-localized to these cells when antiserum to RII (Fig. 7A) or RIV (Fig. 7D) was used. This was confirmed by the cyan coloration of all of the cells in the image overlays (Fig. 7C and 7F). Controls, from which the antiserum to RII or RIV was omitted, failed to show any blue fluorescence with just the secondary antibody present (not shown). In another control reaction, *E. coli* fixed to glass slides stained green with SYTO 9 but showed negligible blue fluorescence from the anti-RII and anti-RIV antibodies used in conjunction with the labeled anti-rabbit second antibody (Fig. S3). Taken together, these results demonstrate that *Mp*AFP is uniformly distributed over the *Marinomonas primoryensis* cell surface and that both the large repetitive RII and the antifreeze domain are exposed to the extracellular environment, consistent with what would be expected for an adhesion protein.

## Discussion

Here we report the purification of wild-type *Mp*AFP, its full amino acid sequence, and show that its tremendous size (ca. 1.5 MDa) is the result of ca. 120 tandem copies of an identical 104-aa repeat that is predicted to form an Ig-like beta-sandwich domain. The region is flanked by one or two non-identical repeats (<65% identity) on each side. All of the other repeats are 100% identical at the DNA level, as shown by sequencing of numerous genomic clones. This suggests that their expansion in *M. primoryensis*, likely by duplication followed by multiple rounds of unequal recombination [27], is a relatively recent event. Each of the ∼120 Ig-like domains is expected to fold as an independent unit, forming a chain as observed for a combinatorial model of mouse cadherin based on electron tomography [28] and X-ray crystallography of five repeats [29]. As the monomers of cadherin and those predicted for *Mp*AFP are spaced approximately 50 Å apart, *Mp*AFP could be over 0.6 µm long, or 20% of the length of the cell. This implies that extreme length is a necessary property of this protein.

The only portions of *Mp*AFP that showed high sequence similarity to proteins in the PDB database were the RTX repeats of RIV and RV. Thus, it was a helpful development to find such convincing structural homologs through the Phyre2 analyses. The different immunoglobulin (Ig)-like domains found in RI through RIII are all similar to the arrays found in well-characterized adhesin proteins like cadherin, fibronectin, and bacterial pili [26], [30], [31], strongly supporting *Mp*AFP's role in adhesion. *Mp*AFP is particularly similar to some large adhesion proteins (LAPs) from the RTX family such as *Pseudomonas putida* LapA and LapF [32], [33], [34]. Not only do the individual regions of *Mp*AFP show structural similarity to the domains of these adhesion molecules, but *Mp*AFP bears an overall domain architecture that matches the arrangement seen in these LAPs (Fig. 5A). This arrangement is characterized by an extremely large repetitive region of Ig-like folds near the N terminus (RII) followed by a non-repetitive region (RIII) and several RTX repeats near to the C terminus (RIV and RV). Intriguingly, no ice-binding characteristics (parallel arrays of Thr and Asx) were present in the RTX repeats of these other adhesion proteins, suggesting *Mp*AFP has divergently evolved specifically for ice binding (Fig. 5B). A comparison of the Weblogo plots for the RTX repeats suggests that the transition to an ice-binding role is relatively minor and involves conversion of the third residue to Thr. The fifth residue already has a high incidence of Asx. The only other difference of note is the lower incidence of Gly in position 1 in the AFP.

A second commonality among these LAPs is that they are all secreted via the TISS. The RTX-like repeats of RIV participate in ice-binding, and it is not clear if they can also serve in a secretion role. However the RTX repeats of RV are likely to act as accessory secretion signals, similar to the C-terminal RTX-repeats in adenylate cyclin toxin [35]. Many proteins secreted by the TISS are encoded within operons containing three transport proteins including an ATP-binding-cassette transporter, a membrane fusion protein and an outer membrane protein [23], but these genes are not found immediately upstream or downstream of the *Mp*AFP gene. However, this is not unprecedented because the gene encoding an RTX-containing protein (FrpC) in *Neisseria meningitidis* that is secreted by the TISS is found at a locus distant from the TISS machinery genes [36]. The genome of *M. primoryensis* has not yet been sequenced, but other *Marinomonas* species such as *M. mediterranea MMB-1*, *M. sp. MWYL1* and *M. posidonica*, do possess TISS genes that do not form an operon with any RTX proteins, suggesting that they are also supplied in *cis*. Therefore we suggest that the RTX-like repeats of RV mediate *Mp*AFP secretion via a TISS that is encoded on a separate operon.

The extreme size of *Mp*AFP makes it one of the largest known proteins and over 100 times larger than a typical AFP, which raises doubts about its previously assumed role as an AFP [8]. Antifreeze activity is a function of AFP concentration [14] and fishes that use AFPs for freeze avoidance accumulate millimolar concentrations of freely-diffusible AFP in their blood and interstitial fluids. It is highly unlikely that these concentrations could be attained with a protein the size of *Mp*AFP. If the only role of *Mp*AFP was to prevent ice growth or inhibit the recrystallization of ice it is likely that natural selection would have led to separation of the AFP portion from *Mp*AFP and its subsequent overexpression. From another perspective, it seems improbable that such a small portion of *Mp*AFP (RIV is ∼2% of the total mass of the protein) determines the protein's function. The crucial role likely resides in RII because it makes up 90% of the mass.

*M. primoryensis* was isolated from Ace Lake in Antarctica [37]. The water layers in this lake do not mix, so oxygen content decreases with depth [38]. *M. primoryensis* is strictly aerobic [39], making it likely that it dwells near the surface of the lake for access to oxygen. The bacteria are denser than the lake water in which they reside and although they possess flagella [39], they would need to expend energy to maintain their position in the water column. The immunodetection results presented herein suggest *Mp*AFP is uniformly localized to the cell surface where it is exposed to the extracellular environment. The ice-binding activity of RIV potentially allows *M. primoryensis* to adhere to ice so that it can remain near the water surface where there is more oxygen and a richer source of nutrients derived from photosynthetic organisms.

The proposed function of *Mp*AFP is reminiscent of the Type I pilus from Gram-negative bacteria, which is a surface adhesin that anchors bacteria to nutrient-rich environments upon binding to its ligand [40]. The majority of the mass of a Type I pilus is composed of 500–3,000 tandem Ig subunits that project away from the cell surface; whereas the adhesive tip of a pilus is used to bind to its ligand. This resembles the role of *Mp*AFP_RIV in adhering to ice.

The binding of a large cell to ice is unlikely to be permanent. We have observed that phage displaying AFP on their coat proteins are not included into a slowly growing mass of ice but appear to be sheared off the surface as ice fronts move laterally by step growth. Given the even larger size of the bacteria it is likely that ice growth will shear off the cells and release them back into the lake prior to rebinding. Such a process would help *M. primoryensis* remain in close proximity to ice on the water surface where oxygen and nutrients are relatively abundant. However, it also appears that *M. primoryensis* can sometimes become encased in ice because the bacterium has been isolated from both sea ice and ice in brackish Antarctic lakes [37], [39]. If it is enveloped, it could potentially be physically damaged by the growing ice [41] or become dehydrated as water migrates to the ice surface [42]. A uniform layer of adhesins decorated with a terminal AFP domain may prevent this and allow the cell to retain a shell of water. Thus, we speculate that the cell-ice interaction could potentially be a way of shielding the bacterium from the harmful effect of ice, and/or a chemotactic (envirotactic) type of response in which the bacteria keep themselves in the upper reaches of lakes for better access to oxygen. This represents a novel function for an antifreeze protein – that of binding an organism to ice. It would give AFPs a third role, distinct from thermal hysteresis (freeze avoidance) and ice recrystallization inhibition (freeze tolerance).

## Supporting Information

Figure S1 **Representative MS/MS spectrum of the IDAGTGNDEIYIK tryptic peptide.** m/z values are shown for the abundant fragments in the mass spectrum above the corresponding peaks. The y series fragments have a charge of +1 and all extend to include the C- terminal lysine displayed on the left-hand side. The sequence of the fragment is displayed at the top of the spectrum.
(TIF)

Figure S2 **Amino acid alignment of** ***Mp*** **AFP_RII with RII-like repeats.** The alignment includes two tandem sequences from the C terminus of RI (RI-1: 184*–287* and RI-2: 288*-394*) and one from the N-terminal sequence of RIII (RIII-1: 209–310). These three sequences are aligned against the 104-aa repeat in RII. The residues shaded black corresponds to residues identical to those in *Mp*AFP_RII, whereas the ones shaded grey mark conservative substitutions.
(TIF)

Figure S3 **Control experiment testing the reactivity of** ***E. coli*** **to anti-RII and anti-RIV polyclonal antibodies.** Immobilized *E. coli* cells were fixed in 1% paraformaldehyde before being incubated with anti-RII (A, B and C) or anti-RIV (D, E and F) polyclonal antibodies, followed by Alexa Fluor 350-conjugated goat anti-rabbit secondary antibody (blue in A and D) and SYTO 9 (green in B and E). Images (C) and (F) are composite images of (A) and (B), and (D) and (E), respectively. A 10-micron scale marker is shown by the white horizontal line in panels C and F.
(TIF)

Table S1 **Comparison of monoisotopic [M+H]^+^ masses and sequences of tryptic peptides from native** ***M. primoryensis*** **AFP to those predicted from the gene sequence.**
(DOCX)

Table S2 **Proteins immediately upstream and downstream of** ***Mp*** **AFP.**
(DOCX)

Text S1 **Bioinformatics analyses of** ***Mp*** **AFP via BLASTp.** When BLAST searches were performed using *Mp*AFP, three sequences from other *Marinomonas* species were detected that are flanked on either side by the same two genes that lie adjacent to the *Mp*AFP gene. The AFP homologues share a similar domain structure in that they posses internal *Mp*AFP_RII-like repeats of ∼100 aas, albeit far fewer than in *Mp*AFP and they also contain C-terminal regions of RTX repeats that are similar to those of *Mp*AFP_RIV and RV (Fig. 5A). However, the RII-like repeats, as well as the bulk of the protein, are variably conserved between species. For example, the only regions where *Mp*AFP and the *M. posidonica* homolog contain over 50% identity are within the first and last ∼150 aa. These homologs also contain variable numbers of RTX repeats near their C-termini that are similar to those from *Mp*AFP_RIV. However, they lack the ice-binding Thr residues in position 3 of the repeat (Fig. 5B i), which suggests these proteins do not bind to ice. Similar domain structures were also detected in other large RTX proteins, including two adhesins from *Pseudomonas putida* (Fig. 5A) that contain many RII-like repeats along with RIV-like RTX repeats (PF00353) that again lack the ice-binding residues (Fig. 5B ii and iii).
(DOCX)
