# Supplementary figures and images for: Re-Evaluation of a Bacterial Antifreeze Protein as an Adhesin with Ice-Binding Activity
Source: PLoS One. 2012 Nov 7;7(11):e48805. doi: 10.1371/journal.pone.0048805 (PMC3492233; doi:10.1371/journal.pone.0048805)

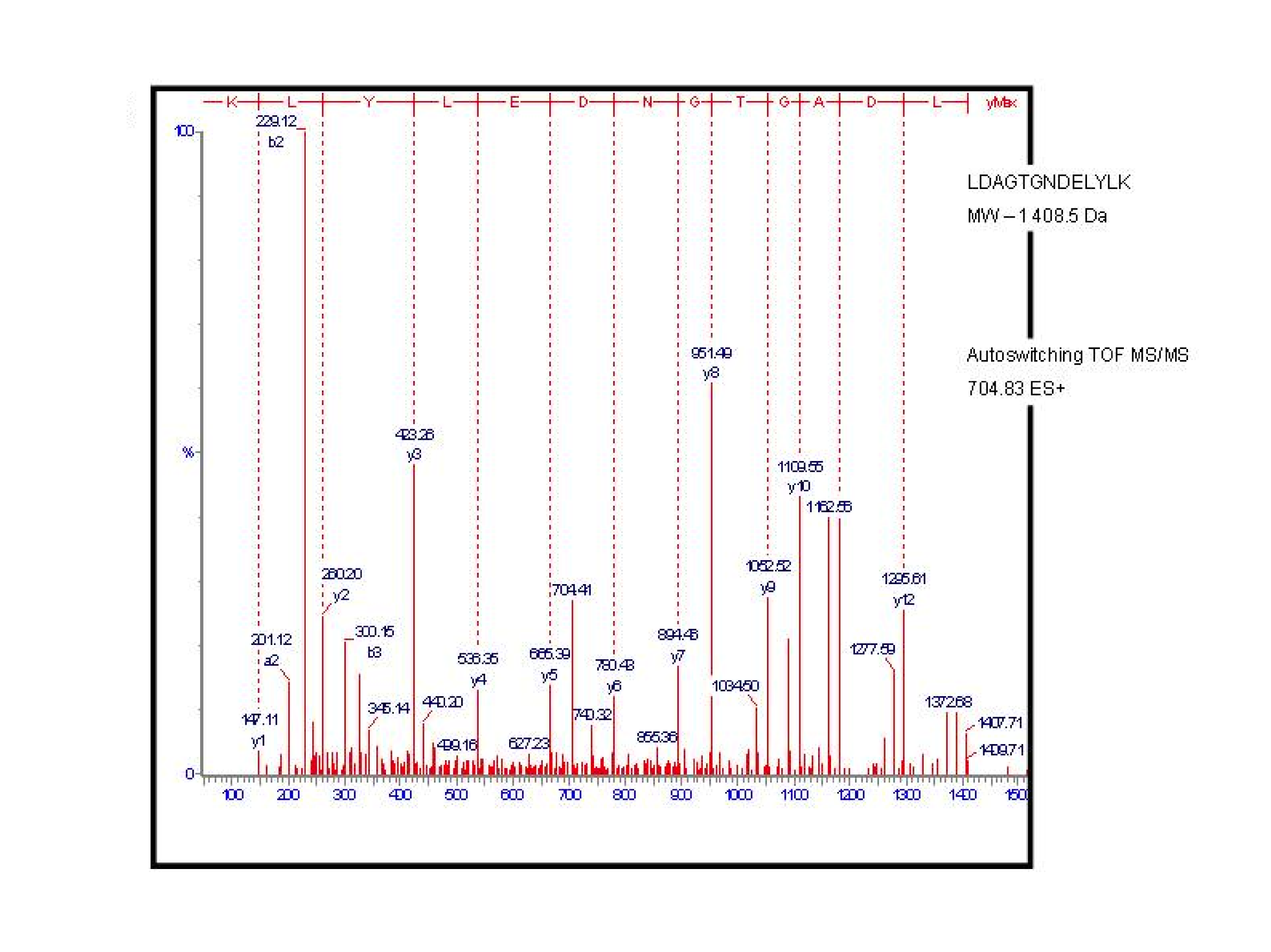

Supplement: Figure S1 — Representative MS/MS spectrum of the IDAGTGNDEIYIK tryptic peptide. m/z values are shown for the abundant fragments in the mass spectrum above the corresponding peaks. The y series fragments have a charge of +1 and all extend to include the C- terminal lysine displayed on the left-hand side. The sequence of the fragment is displayed at the top of the spectrum. (TIF) [file pone.0048805.s001.tif]

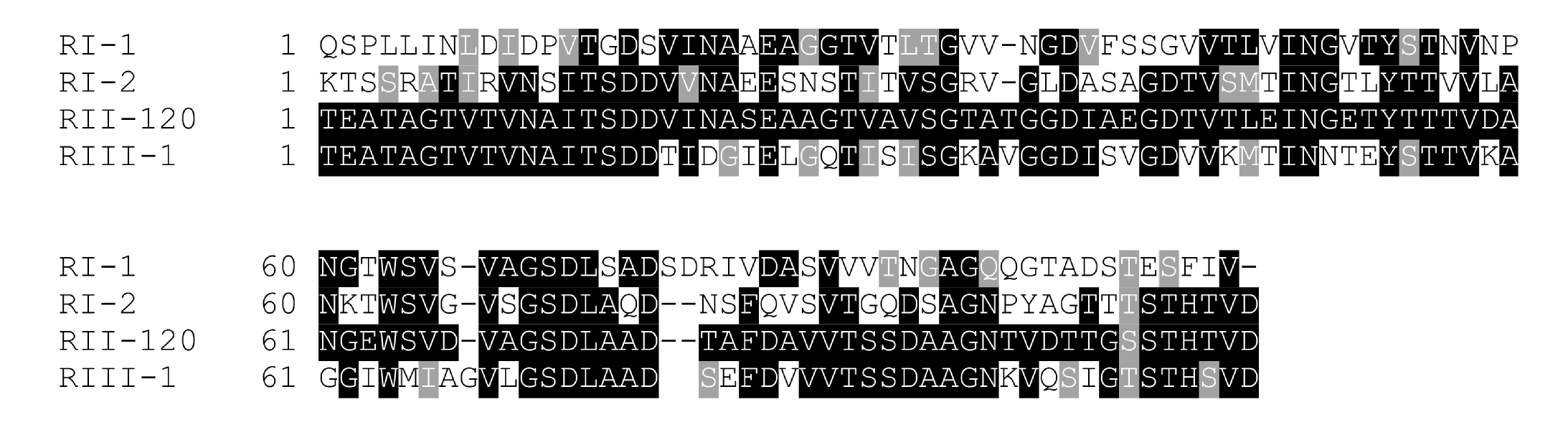

Supplement: Figure S2 — Amino acid alignment of Mp AFP_RII with RII-like repeats. The alignment includes two tandem sequences from the C terminus of RI (RI-1: 184*–287* and RI-2: 288*-394*) and one from the N-terminal sequence of RIII (RIII-1: 209–310). These three sequences are aligned against the 104-aa repeat in RII. The residues shaded black corresponds to residues identical to those in MpAFP_RII, whereas the ones shaded grey mark conservative substitutions. (TIF) [file pone.0048805.s002.tif]

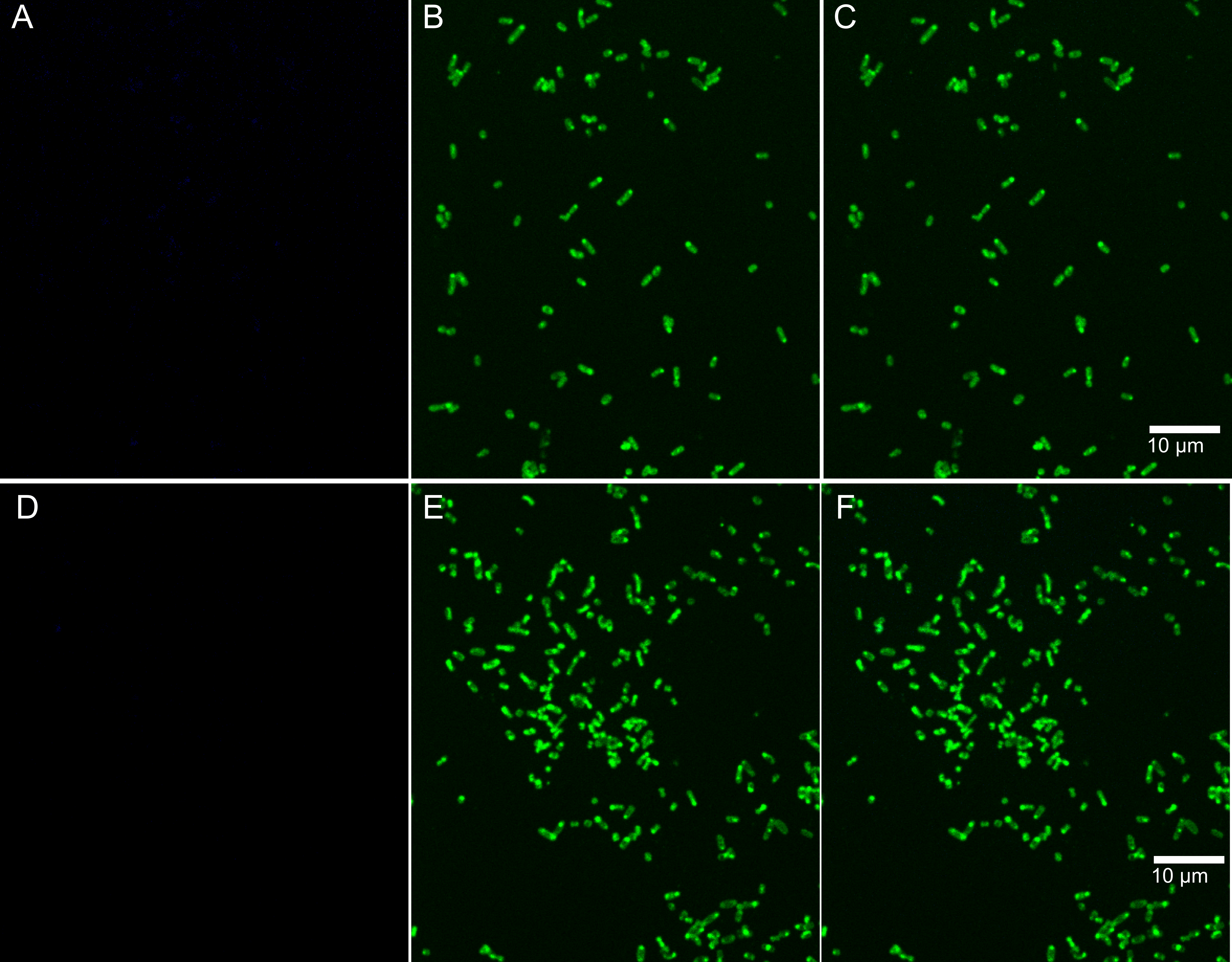

Supplement: Figure S3 — Control experiment testing the reactivity of E. coli to anti-RII and anti-RIV polyclonal antibodies. Immobilized E. coli cells were fixed in 1% paraformaldehyde before being incubated with anti-RII (A, B and C) or anti-RIV (D, E and F) polyclonal antibodies, followed by Alexa Fluor 350-conjugated goat anti-rabbit secondary antibody (blue in A and D) and SYTO 9 (green in B and E). Images (C) and (F) are composite images of (A) and (B), and (D) and (E), respectively. A 10-micron scale marker is shown by the white horizontal line in panels C and F. (TIF) [file pone.0048805.s003.tif]
