# Supplementary material for: Re-Evaluation of a Bacterial Antifreeze Protein as an Adhesin with Ice-Binding Activity
Source: PLoS One. 2012 Nov 7;7(11):e48805. doi: 10.1371/journal.pone.0048805 (PMC3492233; doi:10.1371/journal.pone.0048805)
Supplement: Table S1 — Comparison of monoisotopic [M+H]+ masses and sequences of tryptic peptides from native M. primoryensis AFP to those predicted from the gene sequence. (DOCX) [file pone.0048805.s004.docx]

**Table S1:** **Comparison of monoisotopic [M+H]^+^ masses and sequences of tryptic peptides from native *M. primoryensis* AFP to those predicted from the gene sequence.**

| Pred. Mass^1^ | Exp. Mass^2^ | Charge^3^ | Sequence^4^ | AA Range^5^ | Domain^5^ |
| --- | --- | --- | --- | --- | --- |
| 2172.08 |  |  | MSDNQFPFATLGNAIGFITK | 1-20 | I |
| 1603.79 |  |  | LDGSVTVQSIDGQER | 21-35 | I |
| 817.44 |  |  | VDVSIER | 130-136 | I |
| 1009.49 |  |  | EYSAPSLSR | 172-180 | I |
| 2693.36 | 2693.26 | 2, 3 | IVDASVVVTNGAGQQGTADSTESFIVK | 262-288 | I |
| 2494.19 | 2494.10 | 2 | VNSITSDDVVNAEESNSTITVSGR | 297-320 | I |
| 2811.44 |  |  | VGLDASAGDTVSMTINGTLYTTVVLANK | 321-348 | I |
| 1215.66 | 1215.58 | 2, 1 | **AVGGDISVGDVVK** | 944-956 | III |
| 1501.72 | 1501.66 | 2, 1 | **MTINNTEYSTTVK** | 957-969 | III |
| 720.37 | 720.34 | 1 | YDAPVR | 1066-1071 | III |
| 943.41 |  |  | DHGDAVSDK | 1072-1080 | III |
| 2115.98 | 2115.89 | 2 | DHGD**AVSDKD**SVTVTLEDGR (missed cleavage) | 1072-1091 | III |
| 1191.59 |  |  | DSVTVTLEDGR | 1081-1091 | III |
| 531.29 | 531.26 | 1 | DQIR | 1157-1160 | III |
| 888.42 | 888.39 | 1 | WGNPAESK | 1161-1168 | III |
| 1771.79 | 1771.73 | 2 | **QSGYGFIDND**SNL**EGR** | 1169-1184 | III |
| 1658.85 | 1658.78 | 2 | **DIVTVQNTHVTFER** | 1252-1265 | III |
| 1482.76 | 1482.69 | 2, 1 | DG**DIYTVQIVGFR** | 1266-1278 | III |
| 632.36 | 632.35 | 1 | ALVDSK | 1454-1459 | III |
| 1614.74 | 1614.67 | 2 | **EADATFEAANISYGR** | 1460-1474 | III |
| 1408.70 | 1408.63 | 2, 1 | **IDAGTGNDEIYIK** | 1475-1481 | III |
| 2423.14 | 2423.05 | 2 | LLDNDTDSDNGDTLSVTSISNVK | 1623-1645 | III |
| 2008.02 | 2007.95 | 2 | NGYAYLDAEGIIHFTPVK | 1646-1663 | III |
| 1912.90 | 1912.83 | 2 | GF**AGVATIDYTIE**DGNGG**R** | 1664-1682 | III |
| 663.33 |  |  | GSSDVAK | 1797-1809 | IV |
| 690.38 | 690.32 | 1 | TINASGK | 1887-1893 | IV |
| 1486.76 | 1486.69 | 2 | **VSGNIQGGTGNDIVR** | 1928-1942 | IV |
| 558.29 |  |  | FYTK | 1985-1988 | IV |
| 1252.56 | 1252.50 | 2 | EQYNNNSDLR | 1989-1998 | IV |
| 985.52 | 985.48 | 2, 1 | **VAN**FEHI**R** | 2001-2008 | IV |
| 703.40 | 703.36 | 1 | VSDGVVK | 2009-2015 | IV |
| 1070.48 | 1070.43 | 1 | GSPADFADYK | 2016-2025 | IV |
| 1764.87 | 1764.79 | 2 | DAD**VDAVTQFLTE**NV**K** | 2207-2222 | V |
| 755.40 |  |  | VTDGHVK | 2223-2229 | V |
| 2974.32 |  |  | VGGEDVANFGSDSNFDSNGVDGVTTADSIK | 2230-2259 | V |
| 1642.76 |  |  | VIYNNEEYSINIDG | 2260-2273 | V |
|  | 2165.96 |  | xx**SSFNVVATDNGTGSLTDTK** |  |  |

^1^ Calculated by Peptide Mass [1], [2], only peptides between 0.5 and 3 kDa in mass are shown. ^2^ Determined by LC/ESI-qTOF MS, with an average accuracy and range from expected of -44±40 ppm. ^3^ Observed charge state(s), highest intensity state listed first. ^4^ Matches between the MS/MS and deduced sequences are underlined and bolded (high confidence) or bolded (lower confidence). The undetermined residues of the unidentified sequenced peptide at the bottom of the list are denoted by x. ^5^ Residue numbers and domains as in Figure 3.

References

1. Wilkins MR, Lindskog I, Gasteiger E, Bairoch A, Sanchez JC, et al. (1997) Detailed peptide characterization using PEPTIDEMASS - A World-Wide-Web-accessible tool. Electrophoresis 18: 403-408.

2. Gasteiger E, Gattiker A, Hoogland C, Ivanyi I, Appel RD, et al. (2003) ExPASy: the proteomics server for in-depth protein knowledge and analysis. Nucleic Acids Res 31: 3784-3788.
