# Supplementary material for: Re-Evaluation of a Bacterial Antifreeze Protein as an Adhesin with Ice-Binding Activity
Source: PLoS One. 2012 Nov 7;7(11):e48805. doi: 10.1371/journal.pone.0048805 (PMC3492233; doi:10.1371/journal.pone.0048805)
Supplement: Table S2 — Proteins immediately upstream and downstream of Mp AFP. (DOCX) [file pone.0048805.s005.docx]

**Table S2: proteins immediately upstream and downstream of *Mp*AFP.**

| **Protein** |
| --- |
| 1) Protein of unknown function DUF87 |
| 2) Glutathione synthase |
| 3) Acetyltransferase |
| 4) Phenylserine aldolase |
| 5) Band 7 protein |
| 6) Protein of unknown function DUF107 |
| 7) Permease of the major facilitator superfamily |
| 8) Glyceraldehyde-3-phosphate dehydrogenase |
| 9) Transcriptional regulator, ArsR family protein |
| 10) UspA |
| 11) Putative sulfate permease |
| 12) Peptide methionine sulfoxide reductase |
| 13) Conserved hypothetical protein |
| 14) Cellulose binding, family II, bacterial type: Fibronectin, type III |
| 15) UmuD protein |
| 16) SOS mutagenesis and repair |
| 17) Transposase IS66 |
| 18) Conserved hypothetical protein |
| 19) ORF, no significant matches |
| 20) Conserved hypothetical protein |
