# Supplementary material for: Re-Evaluation of a Bacterial Antifreeze Protein as an Adhesin with Ice-Binding Activity
Source: PLoS One. 2012 Nov 7;7(11):e48805. doi: 10.1371/journal.pone.0048805 (PMC3492233; doi:10.1371/journal.pone.0048805)
Supplement: Text S1 — Bioinformatics analyses of Mp AFP via BLASTp. When BLAST searches were performed using MpAFP, three sequences from other Marinomonas species were detected that are flanked on either side by the same two genes that lie adjacent to the MpAFP gene. The AFP homologues share a similar domain structure in that they posses internal MpAFP_RII-like repeats of ∼100 aas, albeit far fewer than in MpAFP and they also contain C-terminal regions of RTX repeats that are similar to those of MpAFP_RIV and RV (Fig. 5A). However, the RII-like repeats, as well as the bulk of the protein, are variably conserved between species. For example, the only regions where MpAFP and the M. posidonica homolog contain over 50% identity are within the first and last ∼150 aa. These homologs also contain variable numbers of RTX repeats near their C-termini that are similar to those from MpAFP_RIV. However, they lack the ice-binding Thr residues in position 3 of the repeat (Fig. 5B i), which suggests these proteins do not bind to ice. Similar domain structures were also detected in other large RTX proteins, including two adhesins from Pseudomonas putida (Fig. 5A) that contain many RII-like repeats along with RIV-like RTX repeats (PF00353) that again lack the ice-binding residues (Fig. 5B ii and iii). (DOCX) [file pone.0048805.s006.docx]

**Text S1: Bioinformatics analyses of *Mp*AFP via BLASTp.**

When BLAST searches were performed using *Mp*AFP, three sequences from other *Marinomonas* species were detected that are flanked on either side by the same two genes that lie adjacent to the *Mp*AFP gene. The AFP homologues share a similar domain structure in that they posses internal *Mp*AFP_RII-like repeats of ~100 aas, albeit far fewer than in *Mp*AFP and they also contain C-terminal regions of RTX repeats that are similar to those of *Mp*AFP_RIV and RV (Fig. 5A). However, the RII-like repeats, as well as the bulk of the protein, are variably conserved between species. For example, the only regions where *Mp*AFP and the *M. posidonica* homolog contain over 50% identity are within the first and last ~150 aa. These homologs also contain variable numbers of RTX repeats near their C-termini that are similar to those from *Mp*AFP_RIV. However, they lack the ice-binding Thr residues in position 3 of the repeat (Fig. 5B i), which suggests these proteins do not bind to ice. Similar domain structures were also detected in other large RTX proteins, including two adhesins from *Pseudomonas putida* (Fig. 5A) that contain many RII-like repeats along with RIV-like RTX repeats (PF00353) that again lack the ice-binding residues (Fig. 5B ii and iii).
